# Metabolic Engineering of Yeasts: A Key Cell Factory Platform for Advanced Biomanufacturing

**DOI:** 10.3390/jof11120863

**Published:** 2025-12-05

**Authors:** Aiqun Yu, Jiwei Mao, Ning Xu

**Affiliations:** 1 State Key Laboratory of Food Nutrition and Safety, Key Laboratory of Industrial Fermentation Microbiology of the Ministry of Education, Tianjin Key Laboratory of Industrial Microbiology, College of Biotechnology, Tianjin University of Science and Technology, Tianjin 300457, China; 2 Department of Life Sciences, Chalmers University of Technology, SE412 96 Gothenburg, Sweden; 3 Tianjin Institute of Industrial Biotechnology, Chinese Academy of Sciences, Tianjin 300308, China; 4 State Key Laboratory of Engineering Biology for Low-Carbon Manufacturing, Tianjin Institute of Industrial Biotechnology, Chinese Academy of Sciences, Tianjin 300308, China

With mounting concerns over finite fossil fuel reserves and climate change, increasing attention is being paid to an emerging bioeconomy. There is a global consensus about the role of bio-based products, manufactured from renewable raw materials, in ensuring a sustainable bioeconomy [1,2]. Therefore, research into biomanufacturing is developing at a rapid pace, followed by attempts for its industrial application. Microbial cell factories represent a cornerstone of biomanufacturing [3,4,5]. They employ various strategies, technologies, and methods to develop a microbial chassis, which can serve as a super ‘bio-factory’ for the efficient and inexpensive production of chemicals from renewable, low-cost materials. There are four key elements to keep in mind when designing microbial cell factories: materials, chassis, engineering, and products (Figure 1).

Currently employed materials of interest include oil-based feedstocks [6,7,8], cellulosic biomass [9,10,11], and one-carbon compounds [12,13,14]. A deeper understanding of microbial metabolism and continuous advances in metabolic engineering have greatly improved the transformation of raw materials and widened the spectrum of products generated from them by microbial cell factories. However, the poor metabolic performance of most microorganisms when grown on these raw materials, as opposed to glucose, still limits their industrial application.

At present, the most commonly used microbial platforms are bacteria and yeasts; whereas molds, algae, and viruses have found only limited usage. Yeasts, in particular, have many advantages over other microbial sources: they are GRAS organisms, easily cultured with rapid growth, tolerant to various industrial stressors, and genetically tractable with relatively well-developed genetic tools. These characteristics make yeasts particularly attractive for study and engineering in the construction of platform microbial cell factories [15]. At present, some unconventional yeasts have been used as microbial chassis for the production of natural products [16,17,18]. However, more efforts should be invested in the fundamental research on physiological characteristics, metabolic and regulatory information of these unconventional yeasts, as well as their engineering applications in the future.

Utilization of well-suited engineering strategies is a critical factor in achieving high-level product production by microbial cell factories. Metabolic engineering is undoubtedly still of great help in improving cellular processes through redirecting metabolic fluxes. In the past decade, the emergence of new strategies has significantly improved the output of microbial cell factories. Among them, multiscale systems engineering strategy [19,20,21], dynamic regulation technology [22,23,24], and computer-assisted and AI-driven tools [25,26,27] offer great promise and scope for future research.

Finally, advances in metabolic engineering and other technologies have facilitated the development of tailored microbial strains that are capable of producing an expanded range of non-native compounds. A variety of value-added products with complex structure have been produced in different microbial cell factories [28,29,30], demonstrating their potential for the green biosynthesis of industrial products. Although much progress has been made in the use of microbial cell factories for the production of various industrial products, the sub-optimal product titers, yields, and productivities render these platforms far from reaching large-scale commercial exploitation. Meanwhile, it is worth noting that the performance of different microbial chassis may vary substantially even when producing the same compound, a fact that needs to be taken into account and warrants further studies.

The increasing demand for sustainable and efficient biomanufacturing has positioned yeast metabolic engineering at the forefront of industrial biotechnology. The nine contributions collected in this Special Issue exemplify both the conceptual breadth and technical sophistication of current research efforts, encompassing the production of high-value native and non-native metabolites, the valorization of low-cost or renewable feedstocks, the exploitation of non-conventional yeast platforms, as well as the in-depth investigation of yeast stress physiology, epigenetic regulation, and pathogenicity. Liu et al. (Contribution 1) demonstrated that Ca^2+^ can promote the accumulation of the triterpenoid squalene in the yeast *Saccharomyces cerevisiae*. Huang et al. (Contribution 5) demonstrated that sodium butyrate can promote carotenoid synthesis in the yeast *Rhodotorula glutinis.* The study by Maloshenok and co-authors (Contribution 2) assayed the intracellular heterologous expression of PhyD phytase from *Bacillus* species in the yeast *Yarrowia lipolytica*. They successfully overcame aggregation issues and obtained a functionally active product through refolding PhyD phytase using osmolytes (e.g., proline). Zhang and colleagues (Contribution 7) successfully engineered *S. cerevisiae* to de novo produce (2S)-eriodictyol, and the product titer was effectively increased by fine-tuning the metabolism of the (2S)-naringenin synthesis pathway. Wang and colleagues (Contribution 6) successfully engineered *S. cerevisiae* to produce genistein and glycosylation derivatives, and they demonstrated that the systematic engineering approach can increase the product titer in *S. cerevisiae* through the incorporation of a pathway multicopy integration strategy, regulation of the competitive pathway, and enhancement of cofactor availability. An and colleagues (Contribution 3) successfully engineered the industrial rice wine strain *S. cerevisiae* HJ to produce resveratrol, and they demonstrated that the combinatorial metabolic engineering approach effectively improved resveratrol biosynthesis in the industrial *S. cerevisiae* strain through employing a fused-protein methodology and removing feedback inhibition of tyrosine. The study by Deng et al. (Contribution 8) demonstrated that the endoplasmic reticulum–plasma membrane tethering protein Ice2 can control lipid droplet size by controlling intracellular phosphatidylcholine levels in the yeast *Candida albicans.* The study by Du et al. (Contribution 9) demonstrated that the Mec1-Rad53 signaling pathway can regulate DNA damage-induced autophagy and pathogenicity in *C. albicans*. The impact of deleting the DNA damage checkpoint kinase Rad53 on the global transcription profiles and alterations in genes associated with ribosome biogenesis, DNA replication, and cell cycle of *C. albicans* was explored in the work by Zhang et al. (Contribution 4). Overall, these studies showcase innovative strategies and mechanistic insights that are informing the development of robust, high-yielding, and sustainable yeast cell factories. Despite the progress, several critical knowledge gaps persist, including the need for more predictable engineering of non-conventional yeasts, a deeper mechanistic understanding of multi-scale regulatory networks, and improved strategies for metabolic resource allocation and stress tolerance. The convergence of multi-omics analyses, artificial intelligence-driven strain design, and high-throughput engineering platforms is expected to accelerate the construction of intelligent, resilient, and high-performing yeast cell factories, ultimately advancing both fundamental insights into yeast biology and their practical application in sustainable biotechnology.

The Editors of this Special Issue extend their sincere appreciation to all contributing authors, reviewers, and editorial staff, whose valuable efforts were crucial for the successful publication of this Special Issue. It is hoped that researchers in the field of yeast metabolic engineering will work collaboratively to solve the bottlenecks associated with yeast cell factories, significantly improve their ability to synthesize a broader range of target compounds, and promote the wider practical application of metabolically engineered yeast in industrial-scale production.

## Figures and Tables

**Figure 1:**
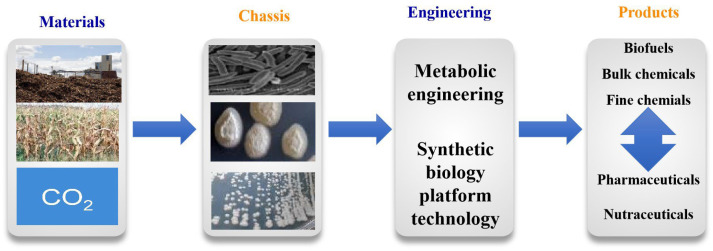
Key elements of microbial cell factories.

## References

[1] Singh R., Gaur A., Soni P., Jain R., Pant G., Kumar D., Kumar G., Shamshuddin S., Mubarak N.M., Dehghani M.H. (2025). A review of biofuels and bioenergy production as a sustainable alternative: Opportunities, challenges and future perspectives. J. Environ. Health Sci. Eng..

[2] Winegar P.H., Astolfi M.C., Holm S.F., Hudson G.A., Keasling J.D. (2025). Advances in the microbial biosynthesis of therapeutic terpenoids. Curr. Opin. Biotechnol..

[3] Yan W., Cao Z., Ding M., Yuan Y. (2022). Design and construction of microbial cell factories based on systems biology. Synth. Syst. Biotechnol..

[4] Sharma A., Yazdani S.S. (2021). Microbial engineering to produce fatty alcohols and alkanes. J. Ind. Microbiol. Biotechnol..

[5] Martinelli L., Nikel P.I. (2019). Breaking the state-of-the-art in the chemical industry with new-to-Nature products via synthetic microbiology. Microb. Biotechnol..

[6] Jiang T., Tan T., Zong Z., Fan D., Wang J., Qiu Y., Teng X., Zhang H.M., Rao C. (2025). Enhancing oil feedstock utilization for high-yield low-carbon polyhydroxyalkanoates industrial bioproduction. Metab. Eng..

[7] Liu Y., Zhang J., Li Q., Wang Z., Cui Z., Su T., Lu X., Qi Q., Hou J. (2022). Engineering *Yarrowia lipolytica* for the sustainable production of β-farnesene from waste oil feedstock. Biotechnol. Biofuels Bioprod..

[8] Abdel-Mawgoud A.M., Markham K.A., Palmer C.M., Liu N., Stephanopoulos G., Alper H.S. (2018). Metabolic engineering in the host *Yarrowia lipolytica*. Metab. Eng..

[9] Li D., Wang F., Zheng X., Zheng Y., Pan X., Li J., Ma X., Yin F., Wang Q. (2025). Lignocellulosic biomass as promising substrate for polyhydroxyalkanoate production: Advances and perspectives. Biotechnol. Adv..

[10] Park J., Park S., Evelina G., Kim S., Jin Y.S., Chi W.J., Kim I.J., Kim S.R. (2024). Metabolic engineering of *Komagataella phaffii* for xylose utilization from cellulosic biomass. Molecules.

[11] Jayakody L.N., Chinmoy B., Turner T.L. (2022). Trends in valorization of highly-toxic lignocellulosic biomass derived-compounds via engineered microbes. Bioresour. Technol..

[12] Fan D., Yuan Z., Tang H., Ren P., Han S. (2025). Metabolic engineering of *Pichia pastoris* as an industrial chassis enables biosynthesis of dopamine from methanol. Bioresour. Technol..

[13] Lee J., Yu H.E., Lee S.Y. (2025). Metabolic engineering of microorganisms for carbon dioxide utilization. Curr. Opin. Biotechnol..

[14] Antonovsky N., Gleizer S., Noor E., Zohar Y., Herz E., Barenholz U., Zelcbuch L., Amram S., Wides A., Tepper N. (2016). Sugar synthesis from CO_2_ in *Escherichia coli*. Cell.

[15] Yook S., Alper H.S. (2025). Recent advances in genetic engineering and chemical production in yeast species. FEMS Yeast Res..

[16] Dmytruk K., Semkiv M., Sibirny A. (2025). Glycerol bioconversion to biofuel and value-added products by yeasts. FEMS Yeast Res..

[17] Korka V., Petropoulos A., Ioannidou S.M., Lin C.S.K., Koutinas A., Fickers P. (2025). Harnessing yeasts for sustainable succinic acid production: Advances in metabolic engineering and biorefinery integration. FEMS Yeast Res..

[18] Wang Y., Wang Y., Cui J., Wu C., Yu B., Wang L. (2025). Non-conventional yeasts: Promising cell factories for organic acid bioproduction. Trends Biotechnol..

[19] Celińska E., Zhou Y.J. (2025). Global transcription machinery engineering in *Yarrowia lipolytica*. FEMS Yeast Res..

[20] Wu Z., Gao J., Gao N., Zhao Y., Zhou Y.J. (2025). Engineering chronological lifespan toward a robust yeast cell factory. Proc. Natl. Acad. Sci. USA.

[21] Ye C., Li X., Liu T., Li S., Zhang M., Zhao Y., Cheng J., Yang G., Li P. (2026). Peroxisome engineering in yeast: Advances, challenges, and prospects. Biotechnol. Adv..

[22] Liu L., Ding D., Wang H., Ren X., Lee S.Y., Zhang D. (2025). Balancing cell growth and product synthesis for efficient microbial cell factories. Adv. Sci..

[23] Qin H., Zhou J., Pang A., Huang L., Liu Z., Zheng Y. (2025). Microbial dynamic regulatory tools: Design, applications, and prospects. ACS Synth. Biol..

[24] Lu M., Sha Y., Kumar V., Xu Z., Zhai R., Jin M. (2024). Transcription factor-based biosensor: A molecular-guided approach for advanced biofuel synthesis. Biotechnol. Adv..

[25] Shi S., Chen Y., Nielsen J. (2025). Metabolic engineering of yeast. Annu. Rev. Biophys..

[26] Lu H., Xiao L., Liao W., Yan X., Nielsen J. (2024). Cell factory design with advanced metabolic modelling empowered by artificial intelligence. Metab. Eng..

[27] Ryu G., Kim G.B., Yu T., Lee S.Y. (2023). Deep learning for metabolic pathway design. Metab. Eng..

[28] Glitz C., Dyekjær J.D., Solimando G.M.C., Avila Neto P.M., Rago D., Babaei M., Borodina I. (2025). Recombinant production of amaranthin and other betalain variants with yeast cell factories. Synth. Syst. Biotechnol..

[29] Qi F., Zhang W., Xue Y., Geng C., Jin Z., Li J., Guo Q., Huang X., Lu X. (2022). Microbial production of the plant-derived fungicide physcion. Metab. Eng..

[30] Leibetseder L., Bindics J., Buyel J.F. (2025). Benzylisoquinoline alkaloid production: Moving from crop farming to chemical and biosynthesis. Biotechnol. Adv..

